# Accuracy of deep learning-based AI models for early caries lesion detection: the influence of annotation quality and reference choice

**DOI:** 10.1007/s00784-025-06672-z

**Published:** 2025-12-04

**Authors:** Ricardo E. Gonzalez-Valenzuela, Pascal Mettes, Bruno G. Loos, Henk Marquering, Erwin Berkhout

**Affiliations:** 1 Department of Oral Radiology, Academic Centre for Dentistry Amsterdam (ACTA), University of Amsterdam and Vrije Universiteit Amsterdam, Gustav Mahlerlaan 3004 (Office 4N-73), Amsterdam, Noord-Holland 1081 LA the Netherlands; 2 Department of Biomedical Engineering and Physics, Amsterdam University Medical Center (AUMC), University of Amsterdam and Vrije Universiteit Amsterdam, Meibergdreef 15, Amsterdam, Noord-Holland 1105 AZ The Netherlands; 3 VISlab, Informatics Institute, University of Amsterdam (UvA), Science Park 904, Amsterdam, Noord-Holland 1098 XH Netherlands; 4 Department of Periodontology, Academic Centre for Dentistry Amsterdam (ACTA), University of Amsterdam and Vrije Universiteit Amsterdam, Gustav Mahlerlaan 3004, Amsterdam, Noord-Holland 1081 LA the Netherlands; 5 Department of Radiology and Nuclear Medicine, Amsterdam University Medical Center (AUMC), University of Amsterdam and Vrije Universiteit Amsterdam, Meibergdreef 15, Amsterdam, Noord-Holland 1105 AZ The Netherlands

**Keywords:** Artificial intelligence (AI), Dental caries detection, Proximal caries lesion, Dental radiology, Image annotation, Training sources

## Abstract

**Objectives:**

The objective of this study is to assess how different annotation methods used during AI model training affect the accuracy of early caries lesion detection, and how the choice of the evaluation reference standard leads to significant differences in assessing AI models’ outcomes. Clinical Relevance. AI-based tools for caries detection are becoming common in dentistry. This study shows that how these models are evaluated can significantly impact perceived performance. Clinicians and developers should ensure that evaluation standards are independent and clinically relevant to avoid overestimating AI’s diagnostic abilities and to build trust for real-world use and regulatory approval.

**Methods:**

Multiple AI caries lesion segmentation models were trained on the ACTA-DIRECT dataset using annotations from (1) single dentists, (2) aggregated strategies (majority vote, consensus meetings, STAPLE), and (3) micro-CT-based methods. Model accuracy was evaluated using two approaches: (1) comparison against micro-CT-based annotations and (2) comparison against the training-matched annotations. Statistical significance of differences in model diagnostic accuracy across annotation strategies was assessed using the McNemar test.

**Results:**

There was no statistically significant difference in diagnostic accuracy among AI models when compared to micro-CT-based annotations. However, the diagnostic accuracy was considered statistically significantly higher when the results of the AI models were evaluated with the training-matched annotations.

**Conclusion:**

Our findings indicate a strong influence of reference standards on AI model evaluation. While annotation strategies during training did not significantly affect AI accuracy in caries lesion segmentation, evaluation was subject to bias when models were tested against different reference standards.

**Clinical relevance:**

AI-based tools for caries detection are becoming common in dentistry. This study shows that how these models are evaluated can significantly impact perceived performance. Clinicians and developers should ensure that evaluation standards are independent and clinically relevant to avoid overestimating AI’s diagnostic abilities and to build trust for real-world use and regulatory approval.

**Graphical abstract:**

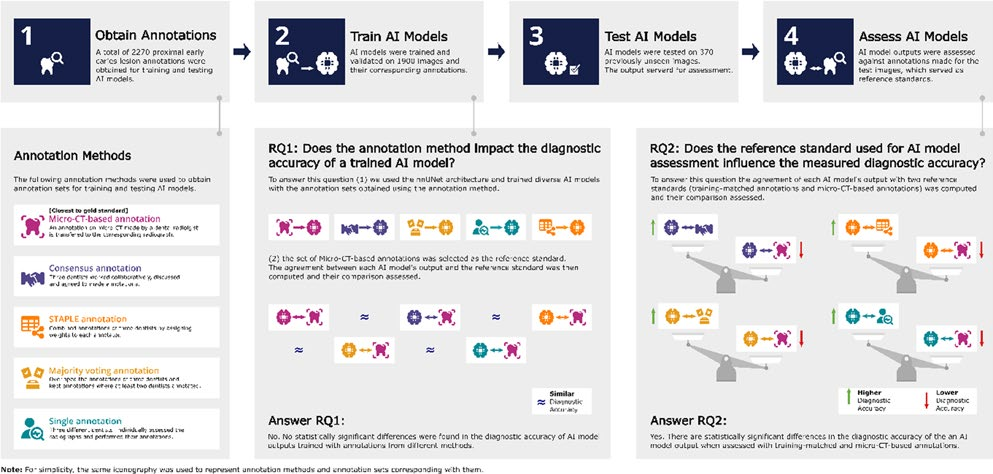

## Introduction

 Bitewing radiographs are indispensable diagnostic resources, aiding dentists in effectively detecting and evaluating proximal caries lesions [1–5]. Bitewing radiographs are particularly effective in revealing advanced stages of caries lesions [6, 7]. However, early stages of caries lesions are usually under-detected [2, 7]. Detection of early proximal caries is valuable for maximizing preventive measures and improving oral health outcomes [1, 8]. Therefore, exploring complementary diagnostic tools holds promise for improved early caries detection [1]. 

Artificial intelligence (AI) algorithms offer significant potential to aid clinical practice and healthcare systems [9, 10]. Several studies have investigated the effectiveness of AI models to detect caries on radiographs, with some demonstrating higher accuracy than dentists [11–15]. These AI models have been trained using caries annotations performed by dentists. This approach raised several concerns. First, annotation noise is a recognized problem arising from variability across annotators and from divergent reference standards which may bias deep learning performance in medical imaging and can substantially affect both model training and evaluation [16]. Second, there are no agreed-upon protocols for annotating lesions, making it difficult to benchmark the performance of AI models and potentially affecting their training [9, 17]. Third, these annotations may be inadequate for accurately identifying caries as the diagnostic quality of dentists in caries diagnostics on radiographs is often limited, particularly due to low sensitivity [9, 10]. Using these traditional dentist annotations as the reference standard for validating AI models may result in incorrect sensitivity and specificity measures [14, 18–20]. Fourth, missing early proximal caries lesions leads to biased results, as an AI model trained with suboptimal manual annotations may exhibit reduced sensitivity for early-stage caries compared to advanced lesions, particularly underperforming in detecting early-stage caries compared to advanced stages [21]. Addressing these concerns could enhance the diagnostic accuracy of AI models, establish a reliable standardized benchmark for their performance, and reduce biases in caries detection outcomes.

While in vivo caries diagnosis results can impact clinical practice, researchers typically define and adhere to their own reference standards [13, 14, 22]. Differences between studies can make it difficult to compare clinical performance. Conversely, caries diagnosis in in vitro studies, while typically employing a histological gold standard for caries assessment, may not capture all the complexities encountered in real-world clinical scenarios [23–25]. Moreover, in studies that employ AI for caries diagnosis, whether in vivo or in vitro, models are usually trained on radiograph annotations provided by dentists, without supplementary validation from methods like histology or micro-CT.

We recently released the ACTA-DIRECT dataset, which provides paired micro-CT images and radiographs from extracted teeth [26]. In a separate study, we developed a method for projecting proximal early caries lesion annotations directly from the micro-CT annotations onto radiographs [27]. These micro-CT-based annotations, which come from high-resolution imaging, offer higher accuracy compared to traditional methods [28]. The micro-CT annotation method addresses the challenges associated with manual dentist-provided annotations, such as low sensitivity for early-stage lesions and high inter- and intra-examiner variability, by providing high-resolution, objective ground truth labels. This makes micro-CT-based annotations more reliable and potentially more suitable for training AI models.

This study aims to evaluate how the choice of annotation method (micro-CT-based vs. traditional dentist-based annotations on radiographs) impacts AI-based lesion segmentation model accuracy. Additionally, we assess whether using a reference standard that matches the training annotations leads to overestimation of the diagnostic assessments of the models’ outcomes, compared to evaluation against a micro-CT-based reference standard.

## Materials & methods

### Data

This study utilized the ACTA-DIRECT dataset, encompassing in vitro imaging studies, including radiographs and micro-CT scans of 179 teeth (totaling 358 proximal surfaces), along with annotations of initial proximal caries. Three dentists, one being a dental radiologist with > 25 years of clinical experience and two being dentists with > 5 years of clinical experience, created lesion annotations on radiographs. A fourth dentist and oral radiologist, also with > 25 years of experience in radiological caries diagnosis and micro-CT imaging, created caries annotations on micro-CT.

Within the ACTA-DIRECT dataset, lesions were categorized by their stage on micro-CT images using the International Caries Classification and Management System (ICCMS) [29]. The ICCMS classifies three stages of initial caries, characterized as radiolucency’s affecting the outer one-half of enamel (RA1), the inner one-half of enamel, potentially extending to the dentine-enamel junction (RA2), and the outer one-third of dentine (RA3).

**Fig. 1:**
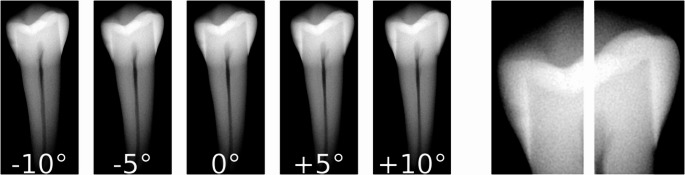
Radiographic series showing tooth orientation variations at −10°, −5°, 0°, + 5°, and + 10°, where 0° represents the buccal side facing the X-ray tube. The image on the right illustrates an example of the cropped proximal-side radiograph utilized for early caries detection in this study

To increase the diversity and representativeness of training data, we expanded the ACTA-DIRECT dataset using the same image acquisition protocol and inclusion/exclusion criteria as in the original dataset [26]. In total, 100 teeth were analyzed, of which 48 were included. For each tooth, four additional radiographs were captured at specific angles (−10°, −5°, + 5°, + 10°), supplementing the existing radiographs taken at 0°, which is the angle for which the buccal side faces the X-ray tube. Since the primary focus in early proximal caries detection is on the proximal sides of the tooth crown, the AI models were trained using cropped sections of these radiographs. As a result, this new version of the ACTA-DIRECT dataset comprises 2270 images (227 teeth × 5 radiographs × 2 proximal sides). Across the 454 proximal radiographs, caries lesion stages were distributed as follows: 286 sound, 72 RA1, 82 RA2, and 14 RA3. Figure 1 illustrates the radiographic orientations and provides an example of a used proximal-side radiograph.

The expanded ACTA-DIRECT dataset (version 2) is accessible in the VU YODA repository at 10.48338/VU01-H5ALYJ.

All radiographs and annotations included in this analysis were complete; no missing data or incomplete annotations were encountered or imputed.

### Proximal caries lesion annotation methodologies

The annotation methodologies were selected based on their use in the literature. All human-based annotations were performed under standardized viewing conditions: dentists worked in a darkened room using a Dell U2419H monitor (24-inch, 1920 × 1080 px, 60 Hz, 8-bit color depth, SDR) calibrated with a SMPTE test pattern, with brightness and contrast set to 75%.

The annotation methodologies are defined as follows to ensure clarity and reproducibility.

- Single Dentist Annotation: A single dentist with over 5 years of clinical experience annotated caries lesions on radiographs.
- Majority Voting Annotation: An annotation is created at pixel level by overlapping the caries lesions annotated by the three dentists and keeping only those pixels marked as lesions by at least two out of the three dentists. Overlapping areas were combined by intersection to ensure lesion consistency.
- Simultaneous Truth and Performance Level Estimation (STAPLE) Annotation: The STAPLE method combines multiple annotations by assigning weights to each annotator. These weights and the final annotation are refined over several iterations based on how much each annotator agrees with the others [26].
- Consensus Annotation: Three dentists with over 5 years of clinical experience worked collaboratively in the same location, discussing and agreeing on the presence and extent of caries lesions in each radiograph to produce a single consensus annotation on radiographs, while viewing a shared display. Discrepancies were resolved through discussion until full agreement was reached.
- Micro-CT-based annotation: A single experienced dentist with over 25 years of clinical experience annotated caries lesions on micro-CT scans, using all projected planes of the scanned tooth for better diagnostics. These annotations were subsequently transferred to the corresponding straight buccal radiographs (0^0^). The transferred annotations were subjected to a quality projection assessment (for more information see Appendix A).

### AI model training and testing workflow for caries segmentation

The following steps were performed for training and testing AI models. The AI models were created using a customized nnUNet architecture to segment caries lesions in radiographic images [26]. All models were implemented using nnUNet v1.7.0 in Python 3.10 and PyTorch 1.13. Further details on the customized architecture are provided in Appendix B. The completed CLAIM checklist can be found in Appendix C.

## Training

We trained and validated our caries segmentation models on a dataset of 1900 images with corresponding caries lesion annotations. We applied cross-validation by dividing the 1900 images into 5 folds, each with a similar distribution of caries lesion stages, comprising on average 240 sound surfaces, 60 RA1, 70 RA2, and 10 RA3 caries lesions. In total, the training and validation sets included 705 caries lesions. To prevent information leakage, we ensured that all images from the same tooth were within a single fold.

The models were trained using the Adam optimizer with an initial learning rate of 1 × 10⁻³, following the default nnUNet learning rate schedule. Training was performed for a maximum of 1,000 epochs, and the model with the best validation loss was saved as the final model. Input images were resampled to [256 × 256] pixels and normalized to zero mean and unit variance per image. Standard nnUNet data augmentation was applied, including random rotations, scaling, elastic deformations, gamma adjustments, and mirroring.

The loss function combined Dice loss and cross-entropy, with default nnUNet class weighting to account for foreground–background imbalance. No additional post-processing beyond the standard nnUNet predicted segmentation handling was applied.

## Testing

We tested the five models on a different set of 370 radiographs, which remained unseen during the training stage. The distribution of the caries lesion stage for the test data matches distribution of the training data. We utilized the nnUNet ensemble and evaluation functions to merge predicted probabilities for caries lesions across the five cross-validation folds.

## Lesion-level mapping and detection rules

Pixelwise probability maps from the AI models were converted into lesion-level detections by applying a fixed probability threshold of *p* > 0.5 (see Appendix D for analyses at thresholds ranging from 0 to 1, with 0.01 increments). Predictions meeting or exceeding this threshold were classified as caries lesions, as this cutoff is commonly adopted in the literature and supports comparability across models and studies. No post-processing steps, such as morphological filtering or smoothing, were applied.

Predicted lesions were matched to reference annotations when at least 25% of their area overlapped. Each proximal surface was assumed to contain a maximum of one lesion. In the few instances (*n* = 3) where two lesions were detected on the same surface, only one was retained, prioritizing the true positive prediction.

Lesion size was not used as an exclusion criterion, as all predicted lesions were of clinically reasonable dimensions after thresholding. This mapping strategy ensured a consistent one-to-one correspondence between predictions and reference annotations, facilitating fair and reproducible lesion-level evaluations across models.

### Assessment

In this study, micro-CT-based caries lesion annotations on radiographs served as the reference standard. To evaluate the accuracy of the AI models, we employed two approaches: (1) the micro-CT evaluation approach, comparing AI models’ lesion annotations with micro-CT-based annotations; (2) the training-matched annotations evaluation approach, comparing AI models’ lesion annotations with the specific annotation type on which they were trained.

Appendix E presents the manual annotation agreement, comparing traditional annotations with micro-CT-based annotations.

### Evaluation metrics

At the lesion level, diagnostic performance was determined as follows: we calculated performance metrics, including sensitivity, specificity, balanced accuracy, positive predictive value (PPV), and negative predictive value (NPV). 95% confidence intervals (CIs) were calculated using the Wilson Score method for binomial proportions.

At the pixel level, the Dice Similarity Coefficient (DSC) was used to quantify the similarity caries lesion regions, only among properly classified caries lesions (true positives, TP).

The overall diagnostic performance for the two approaches was evaluated using receiver operating characteristic (ROC) curves and corresponding area under the curve (AUC) values, accompanied by their respective 95% confidence intervals (CIs) computed using the t-distribution method. This analysis involved varying the decision threshold from 0 to 1 to assess each model’s performance across the full range of possible thresholds.

Additionally, stage-stratified analyses were performed to assess model performance across caries lesion stages (RA1 vs. RA2 - RA3). Sensitivity, specificity, and AUC were computed for each stage following the same evaluation protocol described above, and using micro-CT-based annotations as the reference standard.

### Statistical analysis

In order to address the study’s research questions, multilevel logistic regression analyses were conducted. In all statistical analyses, the fixed effect represented the comparison factor of interest (i.e., type of annotation or reference standard). Random intercepts were specified for the tooth identifier and for the image identifier nested within each tooth. This structure accounted for the dependencies among repeated measures within each tooth and its corresponding images.

Diagnostic outcomes from each AI model were evaluated at the proximal surface level. Agreement with a given reference standard was treated as a binary outcome, reflecting concordance or discordance between the AI prediction and the reference annotation.

To address the first research question “Does the annotation method impact the diagnostic accuracy of a trained AI model?” (RQ1), pairwise comparisons were performed between AI models’ agreements with the micro-CT-based reference standard. A Bonferroni correction was applied to control for multiple comparisons.

To address the second research question “Does reference standard choice impact diagnostic accuracy assessment of AI model?” (RQ2), comparisons were performed individually for each AI model. For each case, the agreement obtained with the micro-CT–based reference standard was compared with that obtained using the training-matched reference standard.

Although both analyses included the same number of observations, the magnitude of the effects led to different statistical power levels, limited for the first analysis and sufficient for the second. A detailed summary of the power and sample size analyses performed for these models is provided in Appendix F.

## Results

### Evaluation metrics

#### Lesion-level evaluation

Table 1 presents the early caries lesion detection performances of AI models compared to the micro-CT-based annotations. The AI model trained with consensus annotations exhibited the highest Sensitivity (0.33) and Balanced Accuracy (0.63). Specificity was consistently high for all AI models, with the model trained on the Expert 2 annotations achieving the highest Specificity (0.97). Both Positive Predictive Value (PPV) and Negative Predictive Value (NPV) were similar for all models, with the model trained on Expert 2 annotations yielding the highest PPV (0.78).

**Table 1:** Performance metrics of AI models compared to the micro-CT annotations as reference standard

AI model trained on	Sensitivity	Specificity	Balanced accuracy	PPV ^a^	NPV ^b^
Micro-CT-based annotations	0.29 (0.22–0.37)	0.94 (0.91–0.97)	0.62 (0.57–0.66)	0.75 (0.62–0.85)	0.70 (0.65–0.75)
Consensus annotations	**0.33 (0.25–0.41)**	0.94 (0.90–0.96)	**0.63 (0.58–0.68)**	0.75 (0.62–0.84)	**0.71 (0.66–0.76)**
STAPLE annotations	0.23 (0.17–0.31)	0.95 (0.91–0.97)	0.59 (0.54–0.64)	0.71 (0.56–0.83)	0.70 (0.64–0.74)
Majority Vote annotations	0.21 (0.15–0.29)	0.96 (0.93–0.98)	0.59 (0.54–0.64)	0.76 (0.60–0.87)	0.69 (0.64–0.74)
Expert 1 annotations	0.17 (0.12–0.24)	0.96 (0.92–0.98)	0.56 (0.51–0.61)	0.70 (0.53–0.83)	0.67 (0.62–0.72)
Expert 2 annotations	0.16 (0.10–0.23)	**0.97 (0.95–0.99)**	0.57 (0.51–0.62)	**0.78 (0.59–0.89)**	0.67 (0.62–0.72)
Expert 3 annotations	0.26 (0.19–0.34)	0.91 (0.86–0.94)	0.58 (0.53–0.63)	0.61 (0.48–0.72)	0.69 (0.64–0.74)

Values in parentheses represent the 95% confidence interval (CI)

Values in bold represent the highest performance for each metric among all annotation methods

^a^PPV, positive predictive value

^b^NPV, negative predictive value

Table 2 shows the performance of AI models for early caries prediction when using the training-matched annotations approach. The models trained with consensus annotations achieved the highest sensitivity (0.44) and balanced accuracy (0.69). The AI models trained using annotations of individual experts demonstrated higher specificity values (0.97). Expert 3 also achieved notable sensitivity (0.4) and balanced accuracy (0.69) values.

**Table 2:** Performance metrics of AI models under the training-matched annotations evaluation approach

AI model trained on	Sensitivity	Specificity	Balanced accuracy	PPV ^a^	NPV ^b^
Micro-CT-based annotations	0.29 (0.22–0.37)	0.94 (0.91–0.97)	0.62 (0.57–0.66)	0.75 (0.62–0.85)	0.70 (0.65–0.75)
Consensus annotations	**0.44 (0.35–0.54)**	0.94 (0.91–0.97)	**0.69 (0.65–0.74)**	0.75 (0.62–0.84)	0.82 (0.78–0.86)
STAPLE annotations	0.36 (0.26–0.47)	0.95 (0.92–0.97)	0.65 (0.60–0.70)	0.64 (0.49–0.77)	**0.85 (0.81–0.89)**
Majority Vote annotations	0.33 (0.24–0.45)	0.96 (0.93–0.98)	0.65 (0.60–0.69)	0.68 (0.51–0.80)	0.85 (0.81–0.88)
Expert 1 annotations	0.30 (0.22–0.41)	**0.97 (0.95–0.99)**	0.64 (0.59–0.69)	0.76 (0.59–0.87)	0.83 (0.79–0.87)
Expert 2 annotations	0.25 (0.17–0.36)	**0.97 (0.95–0.99)**	0.61 (0.56–0.66)	0.70 (0.52–0.84)	0.83 (0.79–0.87)
Expert 3 annotations	0.40 (0.32–0.49)	0.97 (0.94–0.99)	0.69 (0.64–0.73)	**0.88 (0.76–0.94)**	0.77 (0.72–0.81)

Values in parentheses represent the 95% confidence interval (CI)

Values in bold represent the highest performance for each metric among all annotation methods

^a^PPV, positive predictive value

^b^NPV, negative predictive value

For a detailed analysis of sensitivity and specificity across varying thresholds, we refer to Appendix D.

#### Pixel-level evaluation (Similarity of caries lesion regions in properly annotated cases)

Table 3 summarizes the DSC, each computed solely for correctly identified lesions (true positives [TP]). Under the micro-CT evaluation approach, the AI model trained on Expert 1 annotations achieved the highest DSC of 0.64 (within 23 correctly identified lesions). In the training-matched annotations evaluation approach, the AI model trained on consensus annotations reached the highest DSC of 0.74 (within 44 correctly identified lesions).

**Table 3:** Comparison of dice similarity coefficients (DSC) across each of the evaluation approaches. Values represent DSC and true-positive (TP) counts in brackets

Annotation type	Micro-CT-based evaluation approach	Training-matched annotations evaluation approach
Micro-CT-based	0.52 (39)	0.52 (39)
Consensus	0.38 (44)	**0.74 (44)**
STAPLE	0.47 (30)	0.70 (27)
Majority Vote	0.48 (28)	0.71 (25)
Expert 1	**0.64 (23)**	0.67 (25)
Expert 2	0.53 (21)	0.67 (19)
Expert 3	0.38 (34)	0.60 (49)

**Fig. 2:**
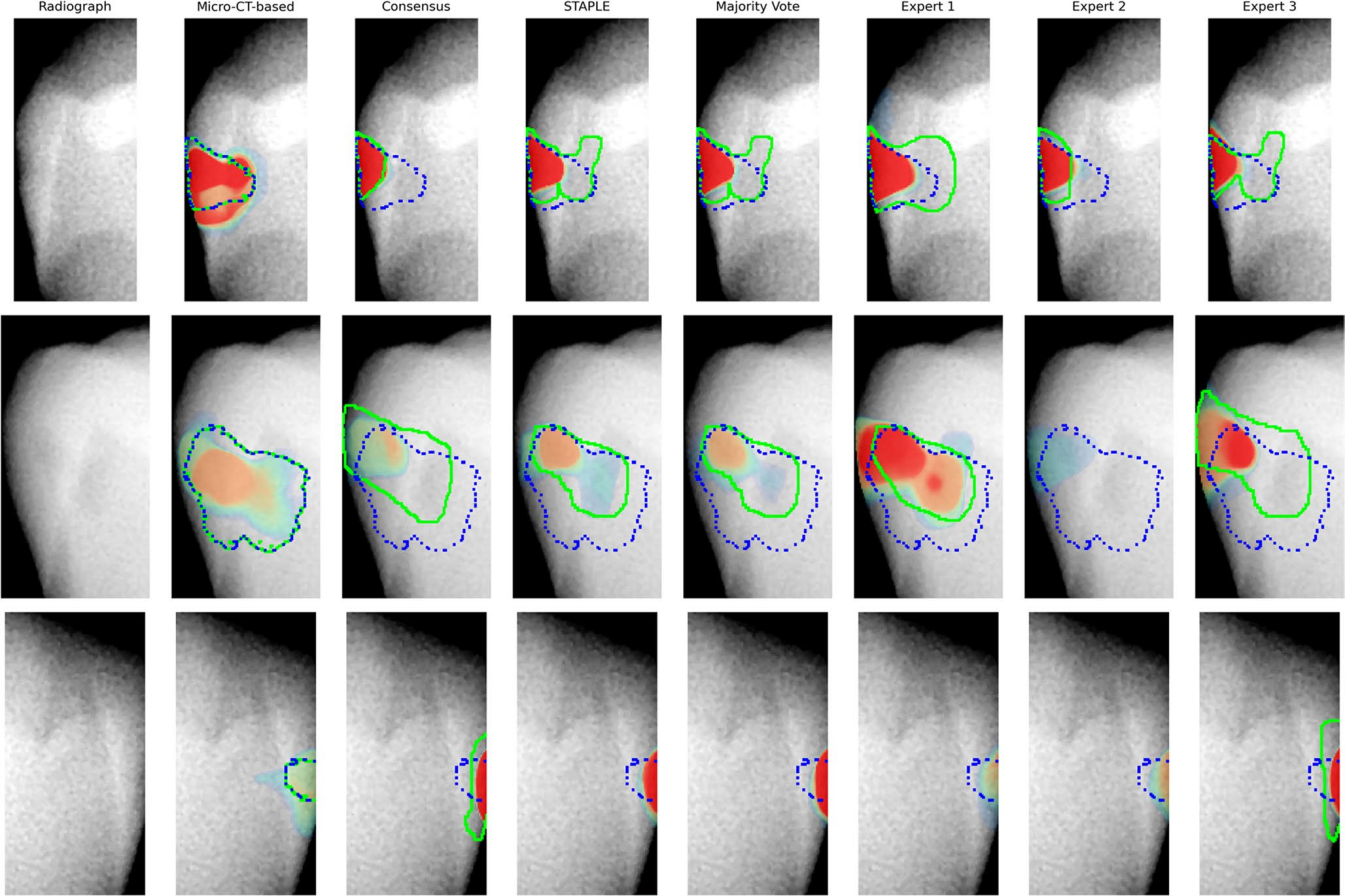
Comparison of AI-predicted caries annotations against different annotation types and the Micro-CT-based reference. The radiograph (first column) is overlaid with AI predictions (heatmaps), individual annotation delineations (green), and the reference standard from Micro-CT-based annotations (dashed blue). The heatmap colors range from purple (≈50% caries probability) to red (≈100% probability)

Figure 2 presents a grid with 3 rows and 8 columns, where each row corresponds to a randomly selected tooth’s proximal surface. The first column displays the original radiograph without annotations, while the remaining seven columns overlay three distinct regions on the same radiograph. The heatmap visualizes the AI model’s prediction corresponding to the annotation type specified in the column title. The color scale ranges from purple to red (purple, blue, green, yellow, orange, red), where purple indicates approximately 50% probability of caries, while red represents a near 100% probability. The solid green contour delineates the actual annotated region according to that annotation type indicated in the column header, whereas the dashed blue contour marks the region derived from the Micro-CT-based annotations, which serve as the reference standard.

For a more detailed analysis of the DSC, refer to Appendix G.

#### Overall performance evaluation

**Fig. 3:**
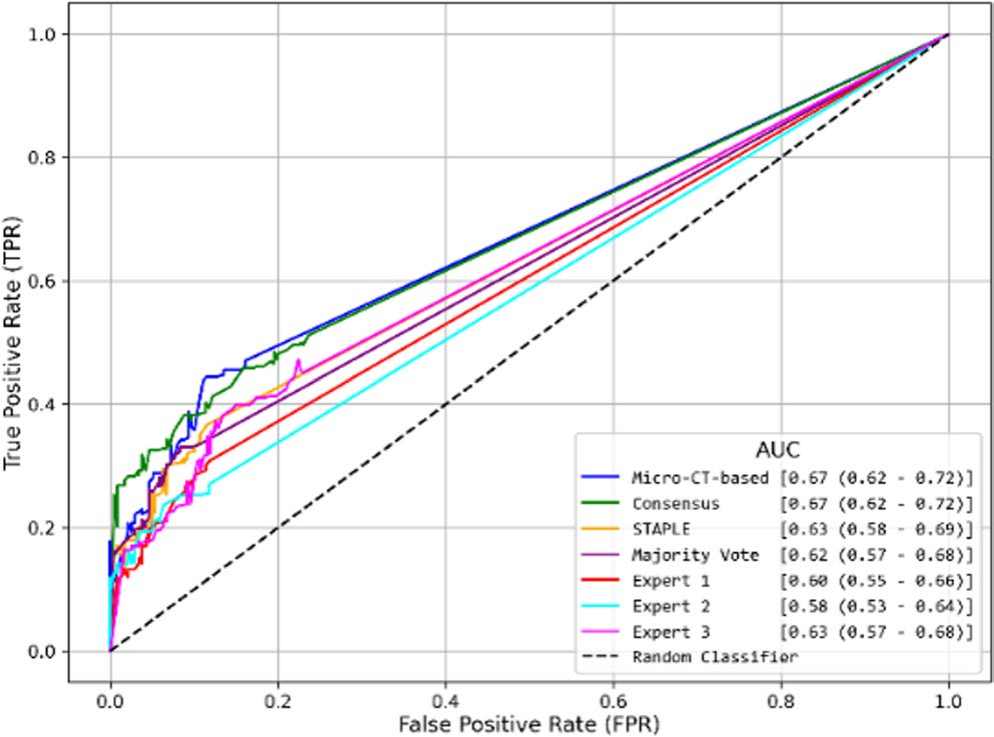
Receiver Operating Characteristic (ROC) curves comparing AI model predictions against micro-CT-based annotations. The legend shows the AUC values with their 95% confidence intervals in parentheses for the various training methods

Figure 3 shows the ROC curves for AI models trained on micro-CT-based and consensus annotations achieving the highest AUC values (0.67), indicating better diagnostic performance compared to the other AI models. The remaining models had quiet similar AUC values (around 0.61).

**Fig. 4:**
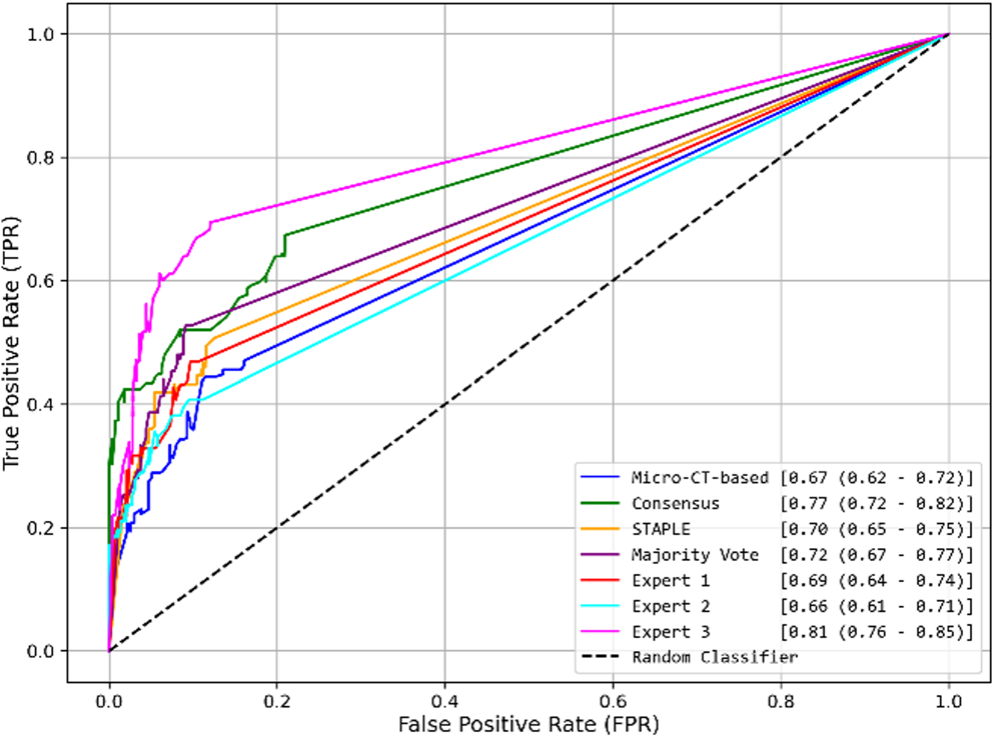
Receiver Operating Characteristic (ROC) curve for the training-matched annotations evaluation approach, comparing AI model predictions against the annotation type used during training. The legend displays the AUC values along with the 95% confidence intervals in parentheses

For the training-matched annotations evaluation approach, Fig. 4 presents the ROC curves for each model. The AI model trained on Expert 3 annotations achieved the highest AUC value (0.81), followed by the model trained on Consensus annotations (0.77). The remaining models show similar AUC values (around 0.69), the model trained on Micro-CT-based annotations exhibits (0.67).

### Stage-stratified performance

As shown in Table 4, the AI models exhibited higher sensitivity for the deeper early caries lesion stages (RA2–RA3) compared to the very early lesion stage (RA1), reflecting the greater difficulty in detecting initial lesions. Specificity remained consistently high across stages and annotation types, generally around 0.92 to 0.98. AUC values for AI models detecting RA1 caries lesion stages ranged from 0.49 to 0.56, while for RA2–RA3 caries lesion stages they ranged from 0.66 to 0.77, indicating that model performance, as measured by AUC, is stage-dependent within the tested approaches.

**Table 4:** Stage-stratified diagnostic performance metrics of AI models trained on different expert annotation types, showing sensitivity, specificity, and AUC with 95% confidence intervals for RA1 proximal caries lesion stage versus RA2–RA3 proximal caries lesion stages, using Micro-CT-based annotations as the reference standard

**AI model trained on annotation type**	**Stage**	**Sensitivity**	**Specificity**	**AUC**
Micro-CT-based	RA1	0.09 (0.02–0.16)	0.94 (0.92–0.97)	**0.56 (0.55–0.56)**
	RA2–RA3	0.47 (0.35–0.59)	0.94 (0.92–0.97)	**0.77 (0.76–0.78.76.78)**
Consensus	RA1	**0.17 (0.08–0.26)**	0.94 (0.91–0.97)	**0.56 (0.55–0.57.55.57)**
	RA2–RA3	**0.47 (0.35–0.59)**	0.94 (0.91–0.97)	**0.77 (0.77–0.78.77.78)**
STAPLE	RA1	0.08 (0.01–0.15)	0.96 (0.94–0.99)	0.52 (0.51–0.53.51.53)
	RA2–RA3	0.37 (0.26–0.49)	0.96 (0.93–0.98)	0.74 (0.73–0.75.73.75)
Majority Vote	RA1	0.10 (0.02–0.17)	0.97 (0.95–0.99)	0.53 (0.52–0.54.52.54)
	RA2–RA3	0.32 (0.21–0.43)	0.97 (0.95–0.99)	0.72 (0.71–0.73.71.73)
Expert 1	RA1	0.06 (0.00–0.12)	0.96 (0.93–0.98)	0.51 (0.51–0.52.51.52)
	RA2–RA3	0.27 (0.17–0.38)	0.96 (0.93–0.98)	0.68 (0.68–0.69.68.69)
Expert 2	RA1	0.08 (0.01–0.14)	**0.98 (0.96–1.00)**	0.49 (0.49–0.50.49.50)
	RA2–RA3	0.23 (0.13–0.33)	**0.97 (0.95–0.99)**	0.66 (0.65–0.67.65.67)
Expert 3	RA1	0.11 (0.03–0.19)	0.92 (0.88–0.95)	0.50 (0.50–0.51.50.51)
	RA2–RA3	0.40 (0.28–0.51)	0.92 (0.88–0.95)	0.76 (0.76–0.77.76.77)

The corresponding confusion matrices from which these stage-stratified metrics were derived are provided in Appendix H.

### Statistical analysis

#### Training annotation impact test (RQ1)

Multilevel logistic regression analyses were conducted for 21 pairwise comparisons of the agreements obtained from AI models trained with different annotation methods. The significance level was adjusted to α = 0.002 (Bonferroni correction for multiple testing). None of the pairwise comparisons of agreements reached statistical significance at the corrected threshold. The lowest p-values were observed when comparing agreements of AI model trained with Consensus and Expert 1 annotations (*p* = 0.005), and Consensus and Expert 1 annotations (*p* = 0.014), Consensus and Expert 2 annotations (*p* = 0.097).

#### Reference Standard Influence Test (RQ2)

Across all AI models, agreements with the two reference standards (micro-CT-based annotations and trained-matching annotations) differed significantly. All comparisons yielded p-values < 0.001. Odds ratios ranged from 2.26 to 4.19, indicating consistently higher agreement when assessed against the training-matched reference standard than when assessed against the micro-CT–based reference standard. The estimated power ranged from 0.87 to 0.98 for all analyses.

## Discussion

Although micro-CT-based annotations provide a more precise reference for in vitro evaluation, our findings did not provide sufficient evidence that their use in training AI models leads to superior diagnostic performance [30]. Evaluation results showed higher diagnostic accuracy when models were assessed against the same type of annotations used during training rather than against high-quality reference data. These findings highlight that measured diagnostic accuracy vary considerably depending on the reference standard applied.

Previous studies have emphasized the value of a gold standard for model validation but have not addressed how varying reference standards impact AI model performance [31]. By incorporating micro-CT-based annotations, our study underscores the importance of high-quality training data in determining whether more detailed information about caries lesions improves model learning, aligning with the broader goal of enhancing AI capabilities to detect early lesions beyond human visual perception [32]. 

Our study presents several strengths. First, it promotes more rigorous data management practices by employing micro-CT-derived information. Second, it provides an expanded version of the ACTA-DIRECT dataset, featuring additional samples, diverse types of annotations, and pre-processed data, making it easily accessible for AI model training and a valuable resource for future research. Third, by demonstrating the varying performance of AI models for caries lesion segmentation trained on different annotation types, we emphasize the importance of a meticulous approach when creating training datasets and recommend that the development of such data should be strongly justified. Fourth, it supports the early exploration of in-vitro data as a foundation for further refinement of AI models to in-vivo settings [33]. Neverthless, we recognize our methodology present some limitations. First, although our findings suggest that an AI model trained on micro-CT-based annotations can highlight certain subtle features in radiographic images that remain imperceptible to dentists, this ability may also work against the model. It might attempt to recognize patterns from micro-CT-based annotations that are not discernible in radiographic images, even with its enhanced capacity to distinguish a broader range of grey intensities, which could negatively impact its training. This limitation could explain why AI models trained on micro-CT-based annotations did not consistently outperform those trained with other annotation methods, as reflected in the results. Second, the study used micro-CT annotations projected onto radiographs as the reference standard. However, these projections are not exact and this slight imprecision might have affected the AI model training. Third, even after expanding the dataset, its size remained a limiting factor, impacting the generalizability of our findings concerning the influence of annotation types on AI model training.

Regarding RQ1, when comparing all models together, no statistically significant differences were observed among the agreements obtained from models trained with different annotation types after Bonferroni correction. Therefore, no definitive conclusion can be drawn in favor of any specific training annotation method. However, when comparing models in pairs (see Appendix F), some showed statistically significant differences, and the performance metrics reveal meaningful tendencies suggesting that differences may exist. Increasing the current sample size would likely better capture the variability needed to represent the different agreement patterns among AI models and allow a more conclusive assessment of RQ1.

Regarding RQ2, the results showed consistent and statistically significant differences in agreement depending on the reference standard used for evaluation. All models achieved higher agreement when assessed against their training-matched reference standard compared to when assessed with the micro-CT–based reference standard. The findings underscore the concern that diagnostic performance reported under currently available reference standards is misleading. Although this study was conducted using in-vitro images, in-vivo evaluations and thus the clinical interpretation of AI performance, could be similarly affected. Even when using Consensus annotations, considered one of the most reliable reference standards currently attainable for in-vivo assessments, performance estimates may still be biased and should be interpreted with caution.

Models trained on micro-CT–based annotations captured finer lesion details but did not necessarily achieve better diagnostic accuracy, likely because such models learn patterns beyond what is perceptible in radiographic images, occasionally leading to misidentifications. In contrast, single-expert models showed greater variability, whereas those trained with aggregated annotations demonstrated more balanced sensitivity and specificity. Interestingly, the model trained with Expert 3 annotations reached the highest sensitivity, though at the cost of reduced specificity—further emphasizing that integrating diverse expert inputs contributes to more robust and stable AI performance.

Comparing AI model performance against micro-CT-based annotations yields several key insights. First, in the micro-CT evaluation and the training-matched annotations evaluation approach, AI models generally exhibited lower sensitivity but higher specificity than their respective training annotations, suggesting a conservative detection tendency. Second, to assess the spatial consistency of AI-segmented caries lesions, we emphasized evaluating only correctly classified lesions. This method provides a clearer understanding of whether AI models are learning meaningful caries lesion patterns, whereas the DSC assessment, as outlined in Appendix G, shows similar results across models due to the predominance of sound regions in dental radiographs. The AI model training using micro-CT-based annotations appeared to better better capture the caries lesions patterns, contributing to one of the highest DSC values among correctly identified lesions. Third, when AI models are assessed against the same annotation type used for their training, their diagnostic performance closely aligns with their respective annotations. However, when micro-CT-based annotations serve as the reference standard, AI model performance is comparable relative to their training annotations, with slight variations—either marginally better or worse. Naturally, the AI model trained on micro-CT-based annotations could not exceed the diagnostic accuracy of micro-CT itself, as it represents the highest available reference standard.

Given that evaluation fairness should prioritize real-world applicability, consensus annotations (as well as aggregated annotations like Majority Voting and STAPLE) appear to be the most suitable for AI model training. Regarding annotation noise, the aggregated annotations clinically reduce inter-annotator variability, improving the reliability of the annotations, while technically, they provide AI models with more consistent training data, enhancing model learning and robustness [34, 35]. However, micro-CT-based annotations serve better as a research benchmark and may still provide benefits as a pre-training set, enabling AI models to better learn caries lesion regions, as demonstrated by the caries lesion similarity assessment. Further fine-tuning could be performed using consensus annotations for in vivo applications, incorporating additional considerations such as adaptation to bitewing images.

To further validate AI models for caries lesion detection, it is essential to explore stage-dependent diagnostic behavior; therefore we stratified the analysis according to caries lesion stage (RA1 vs. RA2–RA3). RA2 and RA3 stages were combined in this analysis as the number of RA3 caries lesion stages in the dataset due to limited representation of RA3 caries lesion stage samples [26]. As shown in Table 4, AI models exhibited higher sensitivity for more advanced lesions (RA2–RA3) while maintaining high specificity across all stages. Among the AI models the ones trained on micro-CT-based annotations and consensus annotations demonstrated superior detection performance for both RA1 and RA2-RA3 caries lesion stages. In general, AI models showed markedly lower sensitivity for RA1 stage lesions, which aligns with the known radiographic limitations in visualizing incipient proximal caries [36]. 

A quantitative comparison with prior caries-AI studies was performed by summarizing sensitivity, specificity, and dataset size across the most relevant literature. Recent systematic reviews show that deep learning models for caries lesion detection on bitewing radiographs report sensitivity values ranging from 0.63 to 0.95 and specificity from 0.86 to 0.99, with dataset sizes varying widely (112 to 8,539 images) [37]. In our study, using the ACTA-DIRECT dataset (370 images), AI models trained with consensus or micro-CT-based annotations achieved sensitivity between 0.29 and 0.44 and specificity between 0.94 and 0.97 when assessed against the micro-CT-based annotations; notably, in our study the specificity was at the upper end of the reported ranges while sensitivity was lower, particularly for very early-stage lesions (RA1: sensitivity ≤ 0.17, RA2/RA3: up to 0.47). This lower sensitivity for early caries lesions is consistent with broader findings that both AI and dentists struggle in this stage, likely due to limitations in radiographic conspicuity and annotation criteria variability [3, 20, 37]. Consequently, while our models demonstrate specificity at the higher end of reported ranges, their lower sensitivity can be attributed to being assessed against a micro-CT-derived reference standard, underscoring the ongoing challenge faced by AI models in detecting early stage caries lesions and highlighting that the use of a high-quality micro-CT reference standard, while essential for accuracy and validation, inherently imposes a more demanding benchmark for performance assessment.

These findings are consistent with recent studies on radiographic and AI-based caries detection performance [37–39]. From a clinical perspective, this distinction is particularly relevant, as detecting caries lesions at the RA1 stage is critical for implementing preventive interventions, yet remains a challenging task for both human observers and AI models. From a technical standpoint, the present results suggest that training with aggregated expert annotations (such as consensus or STAPLE) provides a balanced trade-off between sensitivity and specificity, particularly for lesions at the RA2–RA3 stages.

Interestingly, previous literature reported that AI systems performed not only comparably but sometimes even superior to dentists in detecting early caries lesions [11, 17]. Two main factors may explain this discrepancy. First, although the importance of the reference standard has long been recognized, earlier studies assessing AI models rarely addressed this issue [9, 19, 20]. Only recently have researchers working on deep learning applications for caries detection begun to highlight the limitations of current reference standards for in-vivo research [40]. Second, even when lesion stages are reported, their true extent may be underestimated, as radiographic assessments have been shown to yield smaller apparent lesion depths than those measured histologically [41]. 

Regarding the publicly available datasets, both in-vivo and in-vitro collections address key challenges from complementary perspectives: in-vivo images provide realistic data suitable for training AI models for clinical applications, whereas in-vitro images enable more precise assessments under controlled, high-quality imaging conditions [26, 39]. Unfortunately, these two perspectives remain non-conciliable. An approach to unify clinical applicability and high quality assessment would be to construct a hybrid dataset combining bitewing radiographs annotated with guidance from Cone Beam Computed Tomography (CBCT) inspections, this considering that the CBCT voxel size has been indicated as sufficient for caries detection [26, 42, 43]. A limitation for implementing it is that it would require finding patients who had both bitewing radiographs and artifact-free CBCT scans taken within a short time interval for a clinically justifiable reason, since, due to ethical concerns, radiation dose, cost, and availability, it would not be acceptable to expose patients to CBCT exclusively for caries diagnosis.

Finally, even with a successfully established hybrid dataset, attaining a near or true gold standard still demands careful consideration of several challenging yet essential aspects, including image diversity in quality and source, balanced lesion prevalence, sufficient dataset size, and realistic clinical variability [37]. 

## Conclusion

Our findings indicate a strong influence of reference standards on AI model evaluation for early caries detection. While annotation strategies during training did not significantly affect AI accuracy in caries lesion segmentation, evaluation was subject to bias when models were tested against different reference standards. These results underscore the importance of selecting appropriate reference annotations to ensure reliable AI performance assessment in early caries lesion detection.

## Figures and Tables

**Fig. 5:**
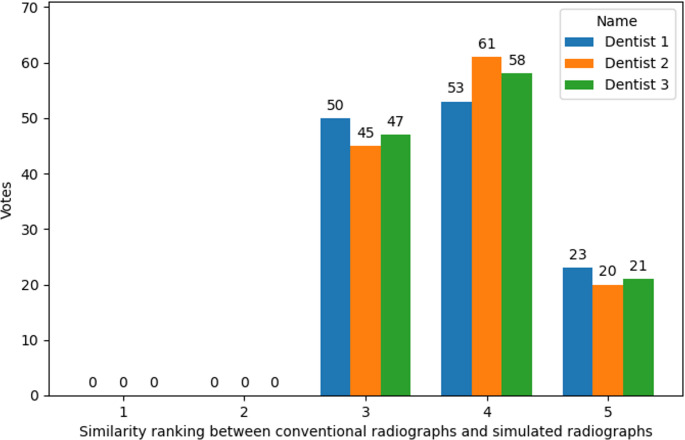
Histogram illustrating the qualitative rankings assigned to tooth projections by three dentists. Each dentist assessed 126 paired micro-CT and radiograph images, with results color-coded by expert

**Fig. 6:**
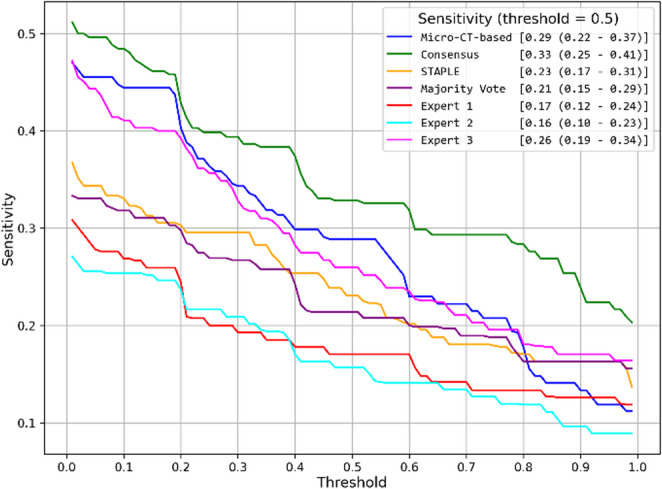
Sensitivity plot across various thresholds for AI models, using micro-CT-based annotations as the reference standard. The sensitivity values at a threshold of 0.5 are shown in the legend, with the values in parentheses indicating the 95% CI

**Fig. 7:**
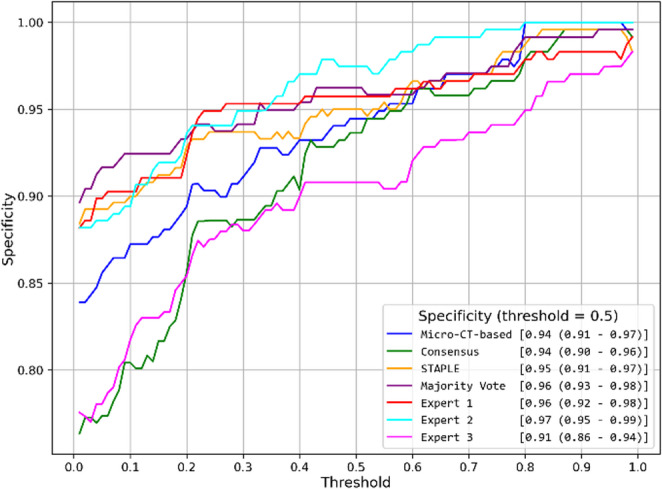
Specificity plot across various thresholds for AI models, using micro-CT-based annotations as the reference standard. The specificity values at a threshold of 0.5 are shown in the legend, with the values in parentheses indicating the 95% CI

**Fig. 8:**
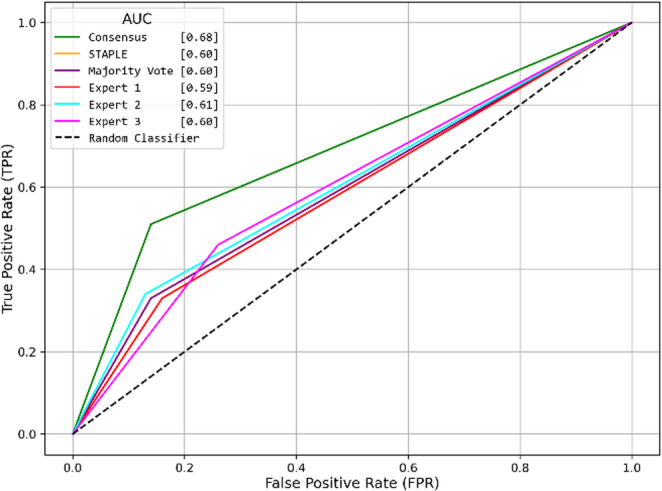
Receiver Operating Characteristic (ROC) curve for the Annotation Agreement Approach. The legend shows the AUC values

**Fig. 9:**
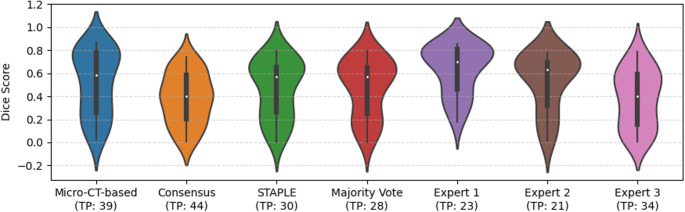
Dice score distribution for true positive (TP) caries lesion predictions by nnUNet models trained on various annotation approaches, compared to micro-CT-based annotations

## Data Availability

The expanded ACTA-DIRECT dataset (version 2) is accessible in the VU YODA repository at [https://doi.org/10.48338/VU01-H5ALYJ] (https://doi.org/10.48338/VU01-H5ALYJ).

## Appendix A – Qualitative projection assessment to ensure adequate caries lesion projection

We conducted a quality evaluation to ensure that micro-CT projections of caries lesions were appropriately annotated, following the criteria established by Gonzalez et al. [27] Three dentists were responsible for evaluating the micro-CT projections to determine their similarity to the radiograph images. The ranking system used is as follows:

- **1**: No similarity
- **2**: Unsuitable similarity
- **3**: Adequate similarity
- **4**: Good similarity
- **5**: Near-perfect similarity

Figure 5 presents the results of our qualitative assessment. Only 126 micro-CT projections were evaluated, as they corresponded to the micro-CT scan in which the 135 caries lesions were identified and were essential for obtaining micro-CT-based annotations of the lesions. Most projections were rated as **4** (good similarity). No projections received a rating of **1** (no similarity) or **2** (unsuitable similarity).

## Appendix B - nnUNet architecture customization

Since the AI model is designed to segment small regions (specifically, caries lesion regions), we aimed to identify a configuration that optimizes performance across all trained nnUNet models for the caries lesion segmentation task. The following changes were made:

1. The**oversample_foreground_percent** attribute of the **nnUNetTrainer** class (in the nnUNetTrainer.py file) was modified to 0.66.
2. The**nnUNet architecture** was adapted with the following modifications:

- **Network Class:** The network class was changed from **ResidualEncoderUNet** to **PlainConvUNet**.
- **Convolutions per Stage:** The number of convolutions per stage in both the encoder and decoder was adjusted. In the encoder, each stage now consists of 2 convolutions (n_conv_per_stage = [2, 2, 2, 2, 2, 2]), compared to the original, which varied ([1, 3, 4, 6, 6, 6]). Similarly, in the decoder, each stage now contains 2 convolutions (n_conv_per_stage_decoder = [2, 2, 2, 2, 2]).
- **Dropout Regularization:** A dropout layer (Dropout2d) with a probability of 0.2 was added to each stage.
- **Batch Size:** The batch size was reduced from 168 to 59 to meet the additional memory requirements.

## Appendix C – Checklist for artificial intelligence in medical imaging (CLAIM)

This appendix contains the Checklist for Artificial Intelligence in Medical Imaging (CLAIM), updated in 2024 by Tejani et al., which provides a framework for comprehensive documentation of AI methods described in the paper and supports reproducibility of the study.

**Table 5:** Checklist for artificial intelligence in medical imaging (CLAIM)

Section : Topic	No.	Item	Included (Yes, No, NA)	Line reference	Comments/Explanation
Title**/** Abstract	1	Identification as a study of AI methodology, specifying the category of technology used (e.g., deep learning)	Yes	1–2, 34–59	Title and abstract explicitly identify the study as AI/deep learning-based.
Abstract	2	Summary of study design, methods, results, and conclusions	Yes	34–59	Abstract clearly summarizes study type, datasets, findings, and implications.
Introduction	3	Scientific and/or clinical background, including the intended use and role of the AI approach	Yes	64–102	Provides clinical background, identifies diagnostic gap, and specifies AI’s intended support role.
	4	Study aims, objectives, and hypotheses	Yes	103–107	Two explicit aims define the research questions and hypotheses.
MethodS: Study Design	5	Prospective or retrospective study	Yes	109–113	Retrospective design using pre-existing datasets; no prospective data collection.
	6	Study goal	Yes	36–39, 222–251	Comparative evaluation of existing AI models under varying annotation/reference conditions.
Methods: Data	7	Data sources	Yes	136–137	ACTA-DIRECT v2 dataset was made publicly available.
	8	Inclusion and exclusion criteria	Yes	122–123	The same setup as for the original dataset was followed.
	9	Data preprocessing	Yes	126–128, 178–182	Radiographs were cropped to proximal regions and pre-processed to standardize inputs for model training.
	10	Selection of data subsets	Yes	171–176, 188–192	Data were divided into cross-validation folds and an independent test set, ensuring balanced lesion distribution and avoiding data leakage.
	11	De-identification methods	N/A	-	The study uses in-vitro data, without any link to identifiable patients.
	12	How missing data were handled	N/A	138–139	There was no missing data.
	13	Image acquisition protocol	Yes	122–123	The same setup as for the original dataset was followed.
Methods: Reference Standard	14	Definition of method(s) used to obtain reference standard	Yes	96–97, 140–165, 603–651	All references standard used were defined in the methodology. Appendix A indicates the procedure to ensure micro-CT-based caries lesion appropriate annotations.
	15	Rationale for choosing the reference standard	Yes	96–97, 104–107	The reason for choosing the reference standards was to evaluate the assessment when compared to different reference standards.
	16	Source of reference standard annotations	Yes	96–97, 140–165	Micro-CT scans were used to create the reference standard closest to gold standard.
	17	Annotation of test set	Yes	140–165	Annotations of all dataset (including test set) were defined in the methodology.
	18	Measures of inter- and intrarater variability of features described by the annotators	Yes	148–160	All aggregated annotation methods were described in the methodology.
Methods: Data Partitions	19	How data were assigned to partitions	Yes	172–176, 189–192	Sample allocation across folds and between the training, validation, and test sets was detailed, with comparable distributions of caries lesion stages maintained in each subset.
	20	Level at which partitions are disjoint	Yes	173–176	Radiographs of proximal surfaces were used, and images from the same proximal surface were kept within the same fold to prevent data leakage.
Methods: Testing Data	21	Intended sample size	Yes	249–251, 772–789	The study includes power and sample size estimations, with full methodological details provided in Appendix F.
Methods: Model	22	Detailed description of model	Yes	167–170, 651–669	This study employed an nnU-Net architecture, and the specific customization procedures are described in Appendix B.
	23	Software libraries, frameworks, and packages	Yes	167–170	Python 3.10, Pytorch 1.13 and nnUNet 1.7.0 are indicate in the study.
	24	Initialization of model parameters	N/A	-	nnU-Net automatically optimized parameters to select the best architecture; custom settings are described in Appendix B.
Methods: Training	25	Details of training approach	N/A	-	Training procedures and hyperparameters followed nnU-Net’s standardized pipeline, with all details and custom settings provided in Appendix B.
	26	Method of selecting the final model	N/A	-	Model selection was handled automatically by nnU-Net’s built-in cross-validation and checkpoint procedures.
	27	Ensembling techniques	N/A	191–192	Ensembling was managed automatically by nnU-Net, which averages predictions from cross-validation folds by default.
Methods: Evaluation	28	Metrics of model performance	Yes	216–231	All evaluation metrics were computed following standard definitions to ensure comparability and reproducibility.
	29	Statistical measures of significance and uncertainty	Yes	232–251	Statistical analyses were performed using multilevel logistic regression with Bonferroni correction; full details are provided in Appendix F. Confidence intervals (95%) were indicated for performance metrics.
	30	Robustness or sensitivity analysis	Yes	191–192	Model performance was evaluated on independent test data after cross-validation, ensuring unbiased assessment.
	31	Methods for explainability or interpretability	Yes	216–231	Performance metrics were computed and interpreted at lesion, pixel, and stratified levels to better understand the research questions.
	32	Evaluation on internal data	Yes	173–175	Assuming internal testing corresponds to validation, model performance was evaluated on a held-out internal test set.
	33	Testing on external data	Yes	169–190	An external test set of 370 radiographs, held out from training and differing in annotation structure, was used to evaluate model performance.
	34	Clinical trial registration	N/A	-	Not a clinical trial
Results: Data	35	Numbers of patients or examinations included and excluded	Yes	123–124	Number of included and excluded teeth was reported.
	36	Demographic and clinical characteristics of cases in each partition	N/A	-	Not applicable to patient demographics; tooth types and lesion-stage distributions are reported and balanced across partitions.
Results: Model Performance	37	Performance metrics and measures of statistical uncertainty	Yes	173–176	Although no patient demographics apply (in-vitro study), tooth types and lesion-stage distributions are reported and balanced across all data partitions.
	38	Estimates of diagnostic performance and their precision	Yes	253–336	Diagnostic performance metrics (Se, Sp, AUC) are reported together with their 95% confidence intervals.
	39	Failure analysis of incorrectly classified cases	Yes	463–474, 801–823	The Discussion section further analyzes these misclassifications and their implications for caries detection performance. Appendix H provides confusion matrices detailing false positives and false negatives.
Discussion	40	Study limitations	Yes	371–386	The discussion explicitly identifies several limitations
	41	Implications for practice, including intended use and/or clinical role	Yes	475–485	The discussion address the importance of reliable reference standards for assessing AI models intended for clinical use.
Other information	42	Provide a reference to the full study protocol or to additional technical details	Yes	633–823	The manuscript provides extensive technical appendices (A–F) detailing qualitative assessment of reference standard, nnUNet architecture customization, detailed performance metrices, and simulation-based power analyses
	43	Statement about the availability of software, trained model, and/or data	Yes	136–137, 168–170	The study uses the publicly available nnU-Net framework and made publicly available the dataset (ACTA-DIRECT v2, DOI), ensuring reproducibility.
	44	Sources of funding and other support; role of funders	Yes	502–506	The study reported the Funding and Conflict of Interest.

The ‘N/A’ option stands for ‘Not Applicable’, as defined in the CLAIM 2024 publication

## Appendix D - Sensitivity and specificity analysis of AI models

This appendix presents an evaluation of the sensitivity and specificity of AI models for caries lesion detection across different classification thresholds. These results highlight the trade-offs between sensitivity and specificity across varying thresholds, providing an overview of model performance trends.

To focus on the most relevant parts of the plots, values at the extreme thresholds (0 and 1) were omitted. At a threshold of 0, sensitivity is always 1 and specificity is 0, whereas at a threshold of 1, sensitivity is 0 and specificity is 1. Since these values are well known and including them would have unnecessarily zoomed out the plots, they were omitted to ensure a clearer visualization of the meaningful variations in model performance.

Sensitivity analysis (see Figure 6) illustrates how effectively each AI model detects caries lesions cases. AI model trained on consensus annotations maintains the highest sensitivity across thresholds, indicating that it detects positive cases more effectively than other models. The micro-CT-based model and Expert 3 also show relatively higher sensitivity, though the former experience a steeper decline as the threshold increases.

Specificity analysis (see Figure 7) examines the AI models’ ability to correctly identify sound caries cases. The AI model trained on the Expert 2 annotations exhibit the highest specificity across most thresholds, suggesting a more stringent classification approach. As thresholds increase, all models show an upward trend in specificity, reflecting a trade-off with sensitivity. This effect is particularly noticeable in models like Consensus, where increased specificity comes at the expense of sensitivity, reducing the likelihood of identifying caries lesion cases at higher thresholds. However, it still outperforms the other AI models at high sensitivity levels.

## Appendix E - Annotation agreement

In this appendix, we present the performance metrics as described in the methodology. The overall diagnostic performance was evaluated using receiver operating characteristic (ROC) curves and corresponding AUC values. However, since this approach provided only a single measurement rather than multiple threshold-based measurements, we were unable to compute 95% confidence intervals (CIs) for the ROC curve. Nevertheless, we include the ROC curve for completeness.

### Evaluation metrics

#### Lesion-level evaluation

Table 6 presents the comparison of the annotation types against micro-CT-based annotations. Consensus annotations achieved the highest values for Sensitivity (0.51), Balanced Accuracy (0.69), Positive Predictive Value (PPV, 0.67), and Negative Predictive Value (NPV, 0.77). The only exception was Specificity, where Expert 2 annotations slightly outperformed Consensus annotations (0.87 vs. 0.86).

**Table 6:** Performance metrics when the annotation agreement approach was used

Type of annotation	Sensitivity	Specificity	Balanced accuracy	PPV ^a^	NPV ^b^
Consensus annotations	**0.51 (0.43–0.60)**	0.86 (0.81–0.90)	**0.69 (0.64–0.73)**	**0.67 (0.57–0.75)**	**0.77 (0.71–0.81)**
STAPLE annotations	0.33 (0.26–0.41)	0.86 (0.82–0.90)	0.60 (0.55–0.65)	0.58 (0.47–0.68)	0.70 (0.64–0.75)
Majority Vote annotations	0.33 (0.26–0.41)	0.86 (0.82–0.90)	0.60 (0.55–0.65)	0.58 (0.47–0.68)	0.70 (0.64–0.75)
Expert 1 annotations	0.33 (0.26–0.42)	0.84 (0.78–0.88)	0.58 (0.53–0.63)	0.53 (0.42–0.63)	0.69 (0.64–0.74)
Expert 2 annotations	0.34 (0.27–0.43)	**0.87 (0.82–0.91)**	0.61 (0.56–0.66)	0.61 (0.49–0.71)	0.70 (0.65–0.75)
Expert 3 annotations	0.46 (0.38–0.54)	0.74 (0.68–0.79)	0.60 (0.55–0.65)	0.49 (0.40–0.58)	0.71 (0.65–0.77)

Values in parentheses represent the 95% confidence interval (CI)

Values in bold represent the highest performance for each metric among all annotation methods

^a^PPV, positive predictive value

^b^NPV, negative predictive value

#### Pixel-level evaluation: Similarity of caries lesion regions in properly annotated cases

Table 7 presents the DSC computed solely for correctly identified lesion regions (true positives[TP]). STAPLE annotations yielded the highest DSC of 0.48. While consensus annotations yielded the lowest DSC of 0.39. It is noteworthy that the DSC for STAPLE was calculated over 44 lesions, compared to 66 for consensus annotations. The micro‐CT-based annotation attains a perfect DSC, as it is compared with itself.

**Table 7:** Comparison of dice similarity coefficients (DSC) across each of the evaluation approaches. Values represent DSC and true-positive (TP) counts in brackets

Annotation type	Annotation agreement approach
Consensus	0.39 (66)
STAPLE	**0.48 (44)**
Majority Vote	0.47 (44)
Expert 1	0.52 (44)
Expert 2	0.44 (46)
Expert 3	0.41 (60)

For a comprehensive analysis of the DSC using its standard evaluation methodology and additional detailed information, refer to Appendix E.

#### Overall performance evaluation

The ROC curves in Figure 8 illustrate the diagnostic performance of each annotation method. The consensus annotation method achieved the highest AUC value (0.68), suggesting the closest alignment with the reference standard. The remaining annotation methods achieved similar AUC values (ranging from 0.59 to 0.61).

## Appendix F – Power and sample size estimation

### Overview

Statistical power was evaluated using Monte Carlo simulation–based methods consistent with the multilevel logistic regression models described in the main text. [44, 45] All analyses were performed in R (version 4.4.0) using the lme4 package for model fitting and the future, furrr, and parallel packages for parallel computation. Binary agreement outcomes were repeatedly simulated under the fitted model parameters, and each simulated dataset was re-analyzed to estimate the proportion of significant results—representing the empirical power. For each condition, 1,000 simulations were run, and statistical significance was assessed at α = 0.05. An odds ratio (OR) of 2.25, previously reported for comparable diagnostic tasks, was used as a benchmark effect size to calibrate the simulations.

Table 8 and Table 9 present the inferential statistics regarding RQ1 and RQ2

**Table 8:** Statistical comparison of AI model performance trained with different annotation methods (Training annotation impact Test, RQ1) – Inferential statistics

Annotation Method 1	Annotation Method 2	*p*-value	Odds Ratio	Empirical Power	Estimated Effective Sample Size	Significant (*p* < 0.05)	Significant (*p* < 0.002)
Consensus	Expert1	0.0052	0.347	0.80	217	Yes	No
Consensus	Expert2	0.0142	0.401	0.69	230	Yes	No
Consensus	Expert3	0.0097	0.331	0.75	225	Yes	No
Consensus	MajorityVote	0.1711	0.570	0.48	250	No	No
Consensus	STAPLE	0.2006	0.616	0.45	181	No	No
Expert1	Expert2	0.4930	1.374	0.43	300	No	No
Expert1	Expert3	0.5818	1.226	0.40	216	No	No
Expert2	Expert3	1.0000	1.000	0.33	238	No	No
MCT	Consensus	0.5482	1.198	0.44	119	No	No
MCT	Expert1	0.0364	0.498	0.61	178	Yes	No
MCT	Expert2	0.0635	0.479	0.58	255	No	No
MCT	Expert3	0.1130	0.594	0.55	191	No	No
MCT	MajorityVote	0.5985	0.831	0.44	221	No	No
MCT	STAPLE	0.5973	0.830	0.44	175	No	No
MajorityVote	Expert1	0.0255	0.291	0.68	282	Yes	No
MajorityVote	Expert2	0.0417	0.225	0.58	414	Yes	No
MajorityVote	Expert3	0.1238	0.488	0.50	239	No	No
STAPLE	Expert1	0.0222	0.282	0.69	288	Yes	No
STAPLE	Expert2	0.0415	0.225	0.57	75	Yes	No
STAPLE	Expert3	0.0901	0.409	0.52	321	No	No
STAPLE	MajorityVote	1.0000	1.000	0.13	407	No	No

**Table 9:** Statistical comparison of AI model assessment with different reference standards (Reference standard influence Test, RQ1) – Inferential statistics

Annotation Method	*p*-value	Odds Ratio	Empirical Power	Significant (*p* < 0.05)
Consensus	0.0001	2.457	0.9	Yes
Expert1	0	3.759	0.97	Yes
Expert2	0	4.191	0.98	Yes
Expert3	0.0001	2.258	0.87	Yes
MajorityVote	0	3.685	0.96	Yes
STAPLE	0	3.463	0.94	Yes

## Appendix G – Dice similarity coefficient (DSC) analysis

Table 10 presents the mean DSC values, which quantify the similarity between the AI models’ caries lesion predictions and the micro-CT-based annotations. Since caries lesions are not present in all samples and are small, making them challenging to segment accurately, the mean DSC for most methods falls within a narrow range of 0.03 to 0.05, suggesting similar performance across models. In contrast, sound surfaces, which dominate the radiographic images and are present in every sample, consistently yield high DSC values of 0.99 across all methods. Consequently, due to the proportion of sound surfaces in the dataset, the overall mean DSC for all AI model predictions remains close to 0.5, regardless of the annotation type used for training.

**Table 10:** Comparison of mean dice similarity coefficient (DSC) values for AI models trained on different types of caries lesion annotation

AI model trained on	Mean DSC for Caries Lesion Segmentation	Mean DSC for Sound Surfaces Segmentation	Mean DSC for Caries Lesion and Sound Surfaces Segmentations
Micro-CT-based annotations	0.05	0.99	0.52
Consensus annotations	0.04	0.99	0.51
STAPLE annotations	0.03	0.99	0.51
Majority Vote annotations	0.04	0.99	0.51
Expert 1 annotations	0.04	0.99	0.51
Expert 2 annotations	0.05	0.99	0.52
Expert 3 annotations	0.04	0.99	0.51

The previously presented data indicated that AI models achieved low and seemingly similar DSC values for caries lesion segmentation, highlighting the need for a more in-depth examination. To address this situation, we present the distribution of Dice scores for properly predicted caries lesion predictions across all the AI models. Table 11 provides deeper insight into the variability and consistency of each annotation strategy, helping to determine which approaches yield more reliable caries lesion segmentations despite the inherent challenges of their small size and limited presence in samples.

**Table 11:** Summary statistics of dice scores for true positive (TP) caries lesion segmentations by AI models trained on different annotation types

AI model trained on	TP	Min	Q1 (25%)	Median (50%)	Q3 (75%)	Max	Mean	Standard Deviation
Micro-CT-based annotations	39	0.03	0.26	0.58	0.79	0.87	0.52	0.28
Consensus annotations	44	0.01	0.2	0.40	0.58	0.74	0.38	0.22
STAPLE annotations	30	0.00	0.26	0.57	0.65	0.8	0.47	0.25
Majority Vote annotations	28	0.00	0.25	0.57	0.66	0.79	0.48	0.25
Expert 1 annotations	23	0.17	0.46	0.70	0.81	0.85	0.64	0.21
Expert 2 annotations	21	0.01	0.32	0.63	0.69	0.78	0.53	0.25
Expert 3 annotations	34	0.01	0.16	0.40	0.59	0.79	0.38	0.25

TP: True Positives

Figure 9 presents the distribution of Dice Similarity Coefficient (DSC) values for true positive (TP) caries lesion predictions across different AI models trained on various annotation types. The AI model trained on Expert 1 annotations exhibits a higher concentration of DSC values around 0.8; however, it is important to note that this model was evaluated on one of the lowest numbers of properly diagnosed caries lesions (TP: 23). In contrast, the model trained on Consensus annotations has the highest number of true positive predictions (TP: 44), but its DSC values are primarily distributed around 0.4, indicating lower segmentation agreement. The AI model trained on Micro-CT-based annotations appears to provide a balanced performance, with a relatively high number of correctly predicted caries lesions (TP: 39) and a predominant distribution of DSC values around 0.8, suggesting both strong predictive accuracy and reliable segmentation performance.

## Appendix H – Confusion matrices by annotation method and lesion stage

This appendix provides detailed confusion matrices for each annotation method and caries lesion stage used in model evaluation. The table summarizes the number of true positives (TP), false positives (FP), true negatives (TN), and false negatives (FN) obtained for models trained on different annotation strategies and evaluated across lesion stages (None, RA1, and RA2–RA3). In the context of caries lesions.

Predicted lesions were matched to reference annotations when at least 25% of their area overlapped. Consequently, false positives (FP) appear in cases where the AI model detected a lesion but the predicted region did not reach the 25% overlap threshold with any reference lesion, or when a lesion was predicted in a surface without a reference lesion.

For the computation of diagnostic performance metrics presented in the main manuscript:

- The values in Table 1 (*Performance metrics of AI models compared to the micro-CT annotations as reference standard*) were obtained by summing the counts from all lesion-stage sections (*None*,*RA1*, and *RA2–RA3*) of Table 12.
- The stage-stratified results in Table 4 (*Stage-stratified diagnostic performance metrics of AI models trained on different expert annotation types, showing sensitivity, specificity, and AUC with 95% confidence intervals for RA1 versus RA2–RA3 proximal caries lesion stages, using Micro-CT-based annotations as the reference standard*) were computed by:
  - RA1: summing the counts from the *None* and *RA1* sections of Table 12, and
  - RA2–RA3: summing the counts from the *None* and *RA2–RA3* sections of Table 12.

**Table 12:** Confusion matrices of AI model predictions for each lesion stage and annotation method

Caries Lesion	Annotation Method	TP	FP	TN	FN
None	Micro-CT-based	0	13	222	0
	Consensus	0	14	221	0
	STAPLE	0	7	228	0
	Majority Vote	0	5	230	0
	Expert 1	0	10	225	0
	Expert 2	0	5	230	0
	Expert 3	0	18	217	0
RA1	Micro-CT-based	6	0	0	59
	Consensus	11	1	0	53
	STAPLE	5	2	0	58
	Majority Vote	6	2	0	57
	Expert 1	4	0	0	61
	Expert 2	5	0	0	60
	Expert 3	7	2	0	56
RA2 - RA3	Micro-CT-based	33	0	0	37
	Consensus	33	0	0	37
	STAPLE	25	3	0	42
	Majority Vote	22	2	0	46
	Expert 1	19	0	0	51
	Expert 2	16	1	0	53
	Expert 3	27	2	0	41

## References

[1] Dayo AF, Wolff MS, Syed AZ, Mupparapu M (2021) Radiology of dental caries. Dent Clin North Am 65(3):427–445. 10.1016/j.cden.2021.02.002 34051924 10.1016/j.cden.2021.02.002

[2] Schwendicke F, Göstemeyer G (2019) Conventional bitewing radiography. In: Zandona AF, Longbottom C (eds) in Detection and assessment of dental caries: a clinical guide. Springer, Cham, p 1. ch. 11 doi: 10.1007/978-3-030-16967-1_11.

[3] Dove SB (2001) Radiographic diagnosis of dental caries. J Dent Educ 65(10):985–990. 10.1002/j.0022-0337.2001.65.10.tb03474.x 11700001

[4] Kidd EA, Pitts NB (1990) A reappraisal of the value of the bitewing radiograph in the diagnosis of posterior approximal caries. Br Dent J 169(7):195–200. 10.1038/sj.bdj.4807325 2223291 10.1038/sj.bdj.4807325

[5] Weiss EI, Tzohart A, Kaffe I, Littner MM, Gelernter I, Eli I (1996) Interpretation of bitewing radiographs. Part 2 evaluation of the size of approximal lesions and need for treatment. J Dent 24(6):385–388. 10.1016/0300-5712(95)00112-3 8990681 10.1016/0300-5712(95)00112-3

[6] Eli I, Weiss EI, Tzohar A, Littner MM, Gelernter I, Kaffe I (1996) Interpretation of bitewing radiographs. Part 1 evaluation of the presence of approximal lesions. J Dent 24(6):379–383. 10.1016/0300-5712(95)00111-5 8990680 10.1016/0300-5712(95)00111-5

[7] Schwendicke F, Tzschoppe M, Paris S (2015) Radiographic caries detection: a systematic review and meta-analysis. J Dent 43(8):924–933. 10.1016/j.jdent.2015.02.009 25724114 10.1016/j.jdent.2015.02.009

[8] Pretty IA, Ekstrand KR (2016) Detection and monitoring of early caries lesions: a review. Eur Arch Paediatr Dent 17(1):13–25. 10.1007/s40368-015-0208-6 26514842 10.1007/s40368-015-0208-6

[9] Schwendicke F, Samek W, Krois J (2020) Artificial intelligence in dentistry: chances and challenges. J Dent Res 99(7):769–774. 10.1177/0022034520915714 32315260 10.1177/0022034520915714 PMC7309354

[10] Naylor CD (2018) On the prospects for a (deep) learning health care system. JAMA J Am Med Assoc 320(11):1099–1100. 10.1001/jama.2018.11103 10.1001/jama.2018.11103 30178068

[11] Cantu AG et al (2020) Detecting caries lesions of different radiographic extension on bitewings using deep learning. J Dent 100:1–8. 10.1016/j.jdent.2020.103425 10.1016/j.jdent.2020.103425 32634466

[12] Talpur S, Azim F, Rashid M, Syed SA, Talpur BA, Khan SJ (2022) Uses of different machine learning algorithms for diagnosis of dental caries. J Healthc Eng. 10.1155/2022/5032435 35399834 10.1155/2022/5032435 PMC8989613

[13] Lee JH, Kim DH, Jeong SN, Choi SH (2018) Detection and diagnosis of dental caries using a deep learning-based convolutional neural network algorithm. J Dent 77(June):106–111. 10.1016/j.jdent.2018.07.015 30056118 10.1016/j.jdent.2018.07.015

[14] Bayraktar Y, Ayan E (2021) Diagnosis of interproximal caries lesions with deep convolutional neural network in digital bitewing radiographs. Clin Oral Investig 26(1):623–632. 10.1007/s00784-021-04040-1 34173051 10.1007/s00784-021-04040-1 PMC8232993

[15] Lee S, il Oh S, Jo J, Kang S, Shin Y, Park Jwon (2021) Deep learning for early dental caries detection in bitewing radiographs. Sci Rep 11(1):16807. 10.1038/s41598-021-96368-7 34413414 10.1038/s41598-021-96368-7 PMC8376948

[16] Wei Y, Deng Y, Sun C, Lin M, Jiang H, Peng Y (2024) Deep learning with noisy labels in medical prediction problems: a scoping review. J Am Med Inform Assoc 31(7):1596–1607 [Online]. Available: http://arxiv.org/abs/2403.13111 10.1093/jamia/ocae108 PMC11187424 38814164

[17] Mohammad-Rahimi H et al (2022) Deep learning for caries detection: a systematic review. J Dent 122:104115. 10.1016/j.jdent.2022.104115 35367318 10.1016/j.jdent.2022.104115

[18] Casalegno F et al (2019) Caries detection with near-infrared transillumination using deep learning. J Dent Res 98(11):1227–1233. 10.1177/0022034519871884 31449759 10.1177/0022034519871884 PMC6761787

[19] Schwendicke F et al (2021) Artificial intelligence in dental research: checklist for authors, reviewers, readers. J Dent 107:103610. 10.1016/j.jdent.2021.103610

[20] Walsh T (2018) Fuzzy gold standards: approaches to handling an imperfect reference standard. J Dent 74(March):S47–S49. 10.1016/j.jdent.2018.04.022 29929589 10.1016/j.jdent.2018.04.022

[21] Oakden-Rayner L, Dunnmon J, Carneiro G, Re C (2020) Hidden stratification causes clinically meaningful failures in machine learning for medical imaging. ACM CHIL 2020 - Proc 2020 ACM Conf Health Inference Learn 151–159. 10.1145/3368555.3384468 10.1145/3368555.3384468 PMC7665161 33196064

[22] Bayrakdar IS et al (2022) Deep-learning approach for caries detection and segmentation on dental bitewing radiographs. Oral Radiol 38(4):468–479. 10.1007/s11282-021-00577-9 34807344 10.1007/s11282-021-00577-9

[23] Syriopoulos K, Sanderink GCH, Velders XL, Van Der Stelt PF (2000) Radiographic detection of approximal caries: a comparison of dental films and digital imaging systems. Dentomaxillofac Radiol 29(5):312–318. 10.1038/sj.dmfr.4600553 10980568 10.1038/sj/dmfr/4600553

[24] Kamburoǧlu K, Kolsuz E, Murat S, Yüksel S, Özen T (2012) Proximal caries detection accuracy using intraoral bitewing radiography, extraoral bitewing radiography and panoramic radiography. Dentomaxillofac Radiol 41(6):450–459. 10.1259/dmfr/30526171 22868296 10.1259/dmfr/30526171 PMC3520392

[25] Devito KL, de Souza Barbosa F, Filho WNF (2008) An artificial multilayer perceptron neural network for diagnosis of proximal dental caries. Oral Surg Oral Med Oral Pathol Oral Radiol Endod 106(6):879–884. 10.1016/j.tripleo.2008.03.002 18718785 10.1016/j.tripleo.2008.03.002

[26] Valenzuela REG, Mettes P, Loos BG, Marquering H, Berkhout E (Oct. 2024) Enhancement of early proximal caries annotations in radiographs: introducing the diagnostic insights for radiographic Early-caries with micro-CT (ACTA-DIRECT) dataset. BMC Oral Health 24(1):1325. 10.1186/s12903-024-05076-x 10.1186/s12903-024-05076-x PMC11526566 39478492

[27] Gonzalez-Valenzuela RE, Vu QTD, Mettes P, Loos BG, Marquering H, Berkhout E (2025) Microtomography to traditional dental radiograph: projecting 3-dimensional initial proximal caries lesion annotations for enhanced radiographic delineation. Dentomaxillofac Radiol 54(4):320–328. 10.1093/dmfr/twae058 39901325 10.1093/dmfr/twae058

[28] Boca C et al (2017) Comparison of micro-CT imaging and histology for approximal caries detection. Sci Rep 7(1):6680. 10.1038/s41598-017-06735-6 28751671 10.1038/s41598-017-06735-6 PMC5532299

[29] Pitts NB, Ismail AI, Martignon S, Ekstrand K, Douglas GVV, Longbottom C (2014) ICCMSTM Quick Reference Guide for Practitioners and Educators. ICCMSTM Resour n/a:1–84

[30] Soviero VM, Leal SC, Silva RC, Azevedo RB (2012) Validity of MicroCT for in vitro detection of proximal carious lesions in primary molars. J Dent 40(1):35–40. 10.1016/j.jdent.2011.09.002 21930181 10.1016/j.jdent.2011.09.002

[31] Boldt J et al (2024) Developing the benchmark: establishing a gold standard for the evaluation of AI caries diagnostics. J Clin Med 13(13):3846. 10.3390/jcm13133846 38999411 10.3390/jcm13133846 PMC11242122

[32] Hung K, Montalvao C, Tanaka R, Kawai T, Bornstein MM (2019) The use and performance of artificial intelligence applications in dental and maxillofacial radiology: a systematic review. Dentomaxillofacial Radiol 49(1). 10.1259/dmfr.20190107 10.1259/dmfr.20190107 PMC6957072 31386555

[33] Rashidi HH, Tran NK, Betts EV, Howell LP, Green R (2019) Artificial intelligence and machine learning in pathology: the present landscape of supervised methods. Acad Pathol. 10.1177/2374289519873088 31523704 10.1177/2374289519873088 PMC6727099

[34] Yu S et al (2020) Robustness study of noisy annotation in deep learning based medical image segmentation. Phys Med Biol. 10.1088/1361-6560/ab99e5 32503027 10.1088/1361-6560/ab99e5 PMC7567130

[35] Shi J, Zhang K, Guo C, Yang Y, Xu Y, Wu J (2024) A survey of label-noise deep learning for medical image analysis. Med Image Anal 95:103166. 10.1016/j.media.2024.103166 38613918 10.1016/j.media.2024.103166

[36] Wenzel A (2014) Radiographic display of carious lesions and cavitation in approximal surfaces: advantages and drawbacks of conventional and advanced modalities. Acta Odontol Scand 72(4):251–264. 10.3109/00016357.2014.888757 24512205 10.3109/00016357.2014.888757

[37] Horvath KSH, Gjerdet NR, Shi X-Q (2025) Advancements in caries diagnostics using bite-wing radiography : a systematic review of deep learning approaches. Caries Res 59:1–32. 10.1159/000546448 40544827 10.1159/000546448 PMC12331221

[38] Ammar N, Kühnisch J (2024) Diagnostic performance of artificial intelligence-aided caries detection on bitewing radiographs: a systematic review and meta-analysis. Jpn Dent Sci Rev 60:128–136. 10.1016/j.jdsr.2024.02.001 38450159 10.1016/j.jdsr.2024.02.001 PMC10917640

[39] Kunt L, Kybic J, Nagyová V, Tichý A (2023) Automatic caries detection in bitewing radiographs: part I—deep learning. Clin Oral Investig 27(12):7463–7471. 10.1007/s00784-023-05335-1 37968358 10.1007/s00784-023-05335-1

[40] Tichý A, Kunt L, Nagyová V, Kybic J (2024) Automatic caries detection in bitewing radiographs—Part II: experimental comparison. Clin Oral Investig 28(2):133. 10.1007/s00784-024-05528-2 38315246 10.1007/s00784-024-05528-2 PMC10844156

[41] Jacobsen JH, Hansen B, Wenzel A, Hintze H (2004) Relationship between histological and radiographic caries lesion depth measured in images from four digital radiography systems. Caries Res 38(1):34–38. 10.1159/000073918 14684975 10.1159/000073918

[42] Qi S, Fu Y, Shan H, Ren G, Chen Y, Zhang Q (2025) Localisation and classification of multi-stage caries on CBCT images with a 3D convolutional neural network. Clin Oral Investig 29(5):246. 10.1007/s00784-025-06325-1 40227550 10.1007/s00784-025-06325-1

[43] Mosavat F, Ahmadi E, Amirfarhangi S, Rafeie N (2023) Evaluation of diagnostic accuracy of CBCT and intraoral radiography for proximal caries detection in the presence of different dental restoration materials. BMC Oral Health 23(1):780. 10.1186/s12903-023-02954-8 37353807 10.1186/s12903-023-02954-8 PMC10290356

[44] Hooper R (2013) Versatile sample-size calculation using simulation. The Stata Journal: Promoting communications on statistics and Stata 13(1):21–38. 10.1177/1536867X1301300103

[45] Johnson PCD, Barry SJE, Ferguson HM, Müller P (2015) Power analysis for generalized linear mixed models in ecology and evolution. Methods Ecol Evol 6(2):133–142. 10.1111/2041-210X.12306 25893088 10.1111/2041-210X.12306 PMC4394709

